# Supplementary material for: Mutant fate in spatially structured populations on graphs: Connecting models to experiments
Source: PLoS Comput Biol. 2024 Sep 6;20(9):e1012424. doi: 10.1371/journal.pcbi.1012424 (PMC11410244; doi:10.1371/journal.pcbi.1012424)
Supplement: S1 Text — We propose a variant of the model that assumes hard selection. In this framework, demes have a carrying capacity that cannot be exceeded during the growth phase. Growth is logistic, and dilutions are performed with a fixed dilution factor. We explain the details of this model, and we justify the choice of parameter values used in S1 Fig. (PDF) [file pcbi.1012424.s001.pdf]

S1 Text for "Mutant fate in spatially structured populations on graphs: connecting models to experiments"

Alia Abbara[1,2], Lisa Pagani[1,2,¤a], Celia García-Pareja[1,2,¤b], Anne-Florence Bitbol[1,2,*]

1 Institute of Bioengineering, School of Life Sciences, École Polytechnique Fédérale de Lausanne (EPFL), Lausanne, Switzerland
2 SIB Swiss Institute of Bioinformatics, Lausanne, Switzerland
¤a Present address: Institute of Integrative Biology, ETH Zürich, Zürich, Switzerland
¤b Present address: Department of Mathematics, School of Engineering Sciences, KTH Royal Institute of Technology, Stockholm, Sweden
*anne-florence.bitbol@epfl.ch

Hard selection variant of the model

In the main text, we focused on a serial dilution model that implements soft selection (see "Model and methods"). Indeed, the total number of migrants sent out from a deme at the dilution step does not depend on its size after growth. In addition, the bottleneck size of each deme has a fixed average value B.

Here, we propose a variant of the model that relies on hard selection. As usual, migration probabilities m_{ij} are defined between any pair of demes, wild-types have fitness $f_W = 1$ and mutants have fitness $f_M = 1 + s$. While all demes are initially of size B, they now also have a carrying capacity K_{sat} that cannot be exceeded during the growth phase. We use a fixed dilution factor d. An elementary step of the model still includes a growth and dilution phase, detailed below.

Logistic growth phase. A phase of logistic growth happens in each deme for a fixed time t. We denote by $M_i(0)$ the number of mutants and by $W_i(0)$ the number of wild-types initially present in deme i. For $\tau \in [0, t]$, these numbers grow following:

$$\frac{dW_i(\tau)}{d\tau} = W_i(\tau) f_W \left(1 - \frac{W_i(\tau) + M_i(\tau)}{K_{sat}}\right), \tag{S1}$$

$$\frac{dM_i(\tau)}{d\tau} = M_i(\tau) f_M \left(1 - \frac{W_i(\tau) + M_i(\tau)}{K_{sat}}\right). \tag{S2}$$

At the end of the growth phase, deme i contains M_i' mutants and W_i' wild-types, and its size $N_i' = M_i' + W_i'$ cannot exceed K_{sat}. The fraction of mutants in deme i at the end of the growth phase is $x_i' = M_i'/N_i'$.

Dilution and migration phase. The dilution phase is similar to the one detailed in "Models and methods", but each deme is now diluted with a fixed factor d. Specifically, the number of mutants (respectively wild-types) that migrate from deme i to deme j is sampled from a binomial distribution with N_i' trials and probability of success $x_i' m_{ij}/d$ (respectively $(1 - x_i')m_{ij}/d$). The mean number of mutants (resp. wild-types) sampled in this way is proportional to M_i' (resp. W_i'). Thus, demes that reach a larger size at the end of the growth phase send out proportionally more individuals. This implements hard selection.

Choice of parameters. In the experiments of Ref. [1], at the end of the growth phase, there was a 30 to 50% reduction in optical density in fully wild-type demes with respect to fully mutant demes [41]. Besides, dilution was performed with a fixed dilution factor $d = 100$ [1]. The initial size of all demes at the beginning of the experiment is $B = 10^7$. For a given value of mutant fitness s, we choose the carrying capacity K_{sat} such that:

- Starting with a bottleneck size $B = 10^7$, the size of a fully wild-type deme increases by a factor 100 during the growth phase. Therefore, in the absence of migrations, deme size would remain equal to B at the next bottleneck.

- A fully mutant deme, initialized at bottleneck size $B = 10^7$, increases in size through successive bottlenecks. After a few steps of the dynamics, it reaches carrying capacity K_{sat} at the end of each growth phase.

- The size reached by a fully wild-type deme at the end of the growth phase is 40% smaller than the carrying capacity, i.e. $100B = 0.6K_{sat}$.

For $s = 0.2$, the corresponding carrying capacity is $K_{sat} \approx 1.9 \times 10^9$ and the growth time is $t \approx 5.3$. For $s = 0.3$, we obtain $K_{sat} \approx 1.8 \times 10^9$ and $t \approx 5.4$. With these parameter choices, we reproduce to the best of our knowledge the conditions of the experiments conducted in [1]. Note however that a more precise match could be obtained if complete growth curves for both the mutant and the wild-type were available in the experimental conditions. Another useful point would be to measure division rate and death rate independently of each other in the presence of antibiotic. This would allow more detailed modeling of antibiotic action.

In Fig. S1, we show the growth of mutant fraction in different structures, in a case matching the top right panel from Fig. 2 in the main text, but in the hard selection variant of the model. Fig. S1 can be directly compared to Fig. 3 from Ref. [1], in the "IN>OUT" setting. As these experiments, we focus on the case of mutants initially introduced in the leaf. Migration rates of $m = 0.01$ for the clique and $m_O = 0.01$ for the star correspond to the "High (1%)" migration intensities in [1]. Migration rates of $m = 10^{-5}$ for the clique and $m_O = 10^{-5}$ for the star correspond to the "Low (0.001%)" case. We recover the same main conclusions in the hard selection variant of our model as those obtained with the soft selection model, presented in the main text. In both cases, the mutant fraction growth displays thresholds for small migration intensities, as demes are successively invaded by mutants. However, the mutant fractions reached at plateaus are different, because in our hard selection model, the bottleneck sizes of the demes depend on their composition. We still obtain a very good agreement between the deterministic model (adapted to include hard selection) and our simulation results. Again, we find that the mutant fraction does not grow faster in the star than in the clique.

Note that the mutant fraction grows faster in experiments from [1] than in our simulations. Taking a larger mutant fitness advantage in our model could account for this difference. As shown in the right panel of Fig. S1, setting $s = 0.3$ accelerates the mutant fraction growth with respect to the left hand panel where $s = 0.2$ (which was chosen to match the measurements of [1]).

References

1. Chakraborty PP, Nemzer LR, Kassen R. Experimental evidence that network topology can accelerate the spread of beneficial mutations. Evol Lett. 2023;7:447–456.
