# Supplementary figures and images for: Mutant fate in spatially structured populations on graphs: Connecting models to experiments
Source: PLoS Comput Biol. 2024 Sep 6;20(9):e1012424. doi: 10.1371/journal.pcbi.1012424 (PMC11410244; doi:10.1371/journal.pcbi.1012424)

**Mutant fitness advantage  $s=0.2$**

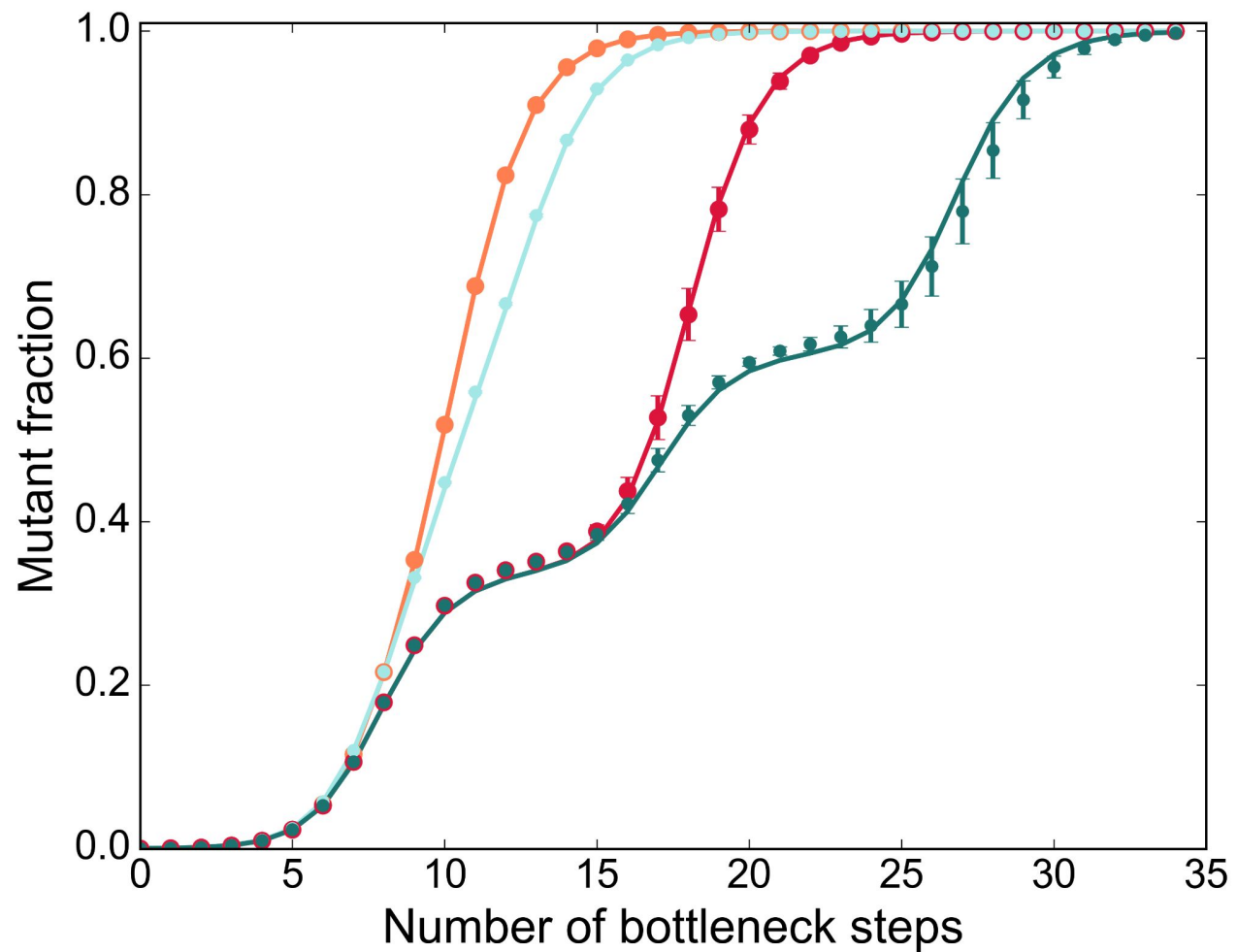

**Mutant fitness advantage  $s=0.3$**

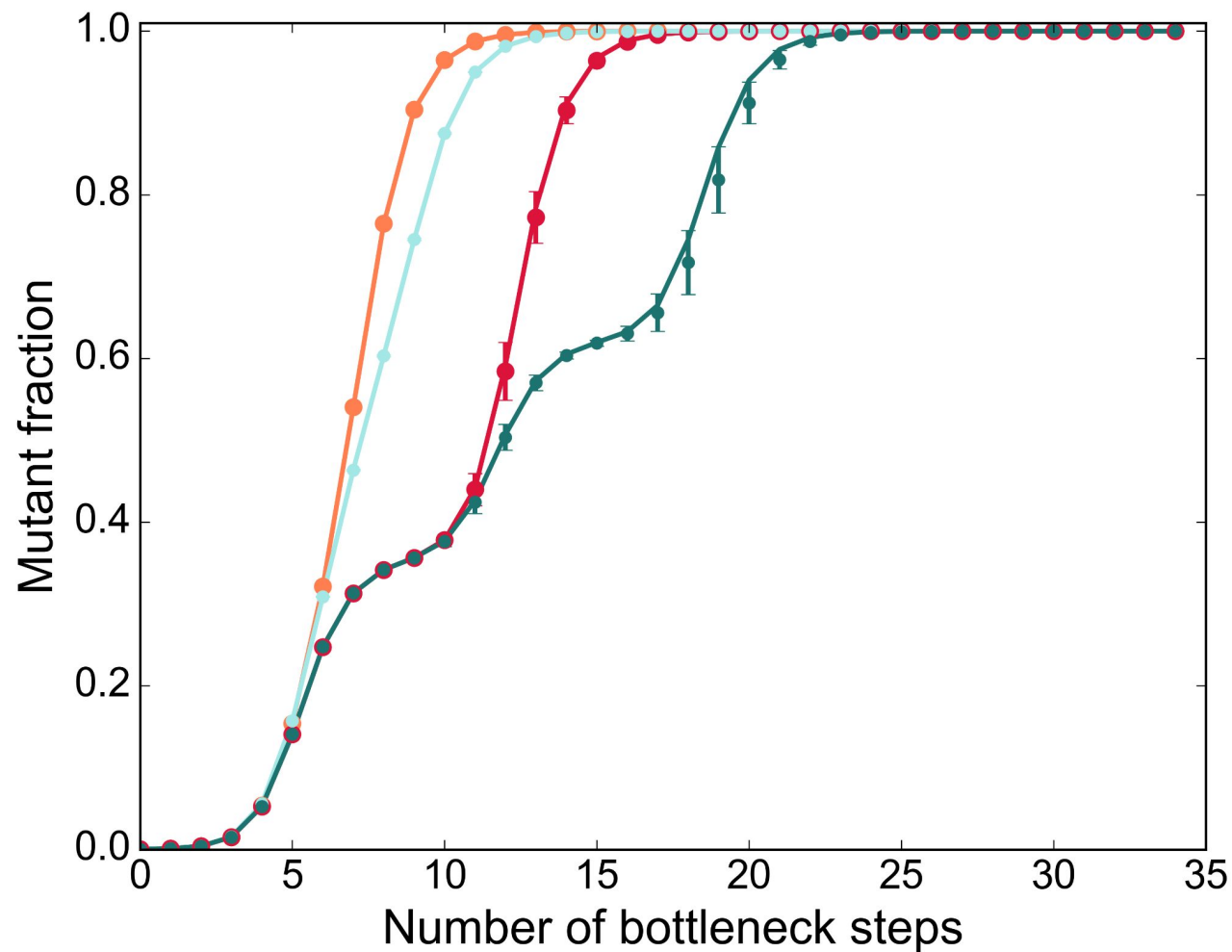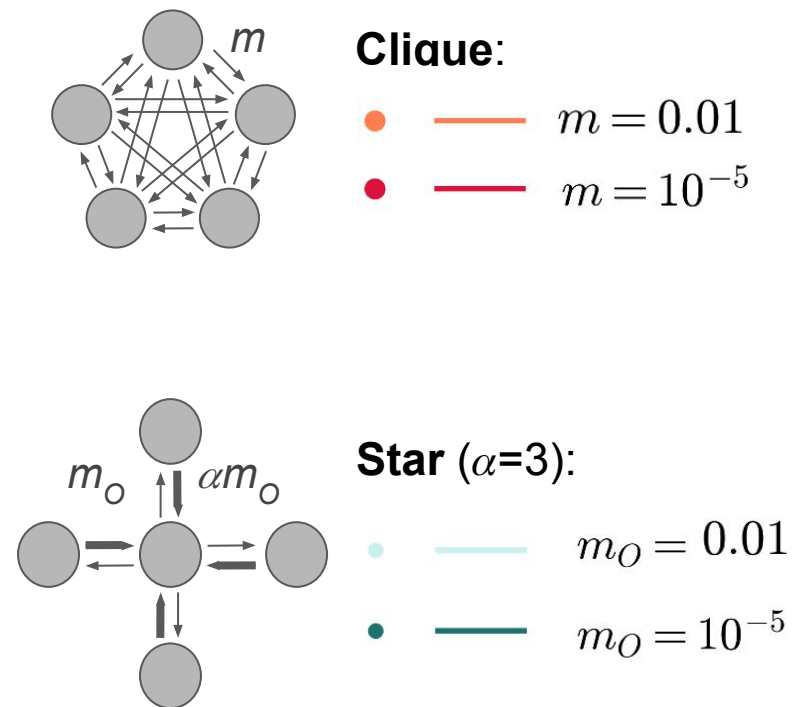

Supplement: S1 Fig — As in [35], we consider a clique and a star with D = 4 demes of initial bottleneck size B = 107, where 104 mutants with fitness advantage s = 0.2 (left) and s = 0.3 (right) are initially placed in one leaf for the star structure, any leaf for the clique. The dilution factor is d = 100. In each panel, the carrying capacity and growth time are chosen to obtain a 40% optical density between fully wild-type demes and fully mutant demes after growth. We consider different migration probabilities m for the clique and mI for the star, satisfying m = mO and α = mI/mO = 3 for the star. Markers are simulation results averaged over 100 trajectories, and error bars report the standard deviation. Note that all trajectories resulted in mutant fixation. Lines show the predictions from our deterministic model, adapted to the hard selection version of the model. (PDF) [file pcbi.1012424.s002.pdf]

**Mutants initially placed in the center**

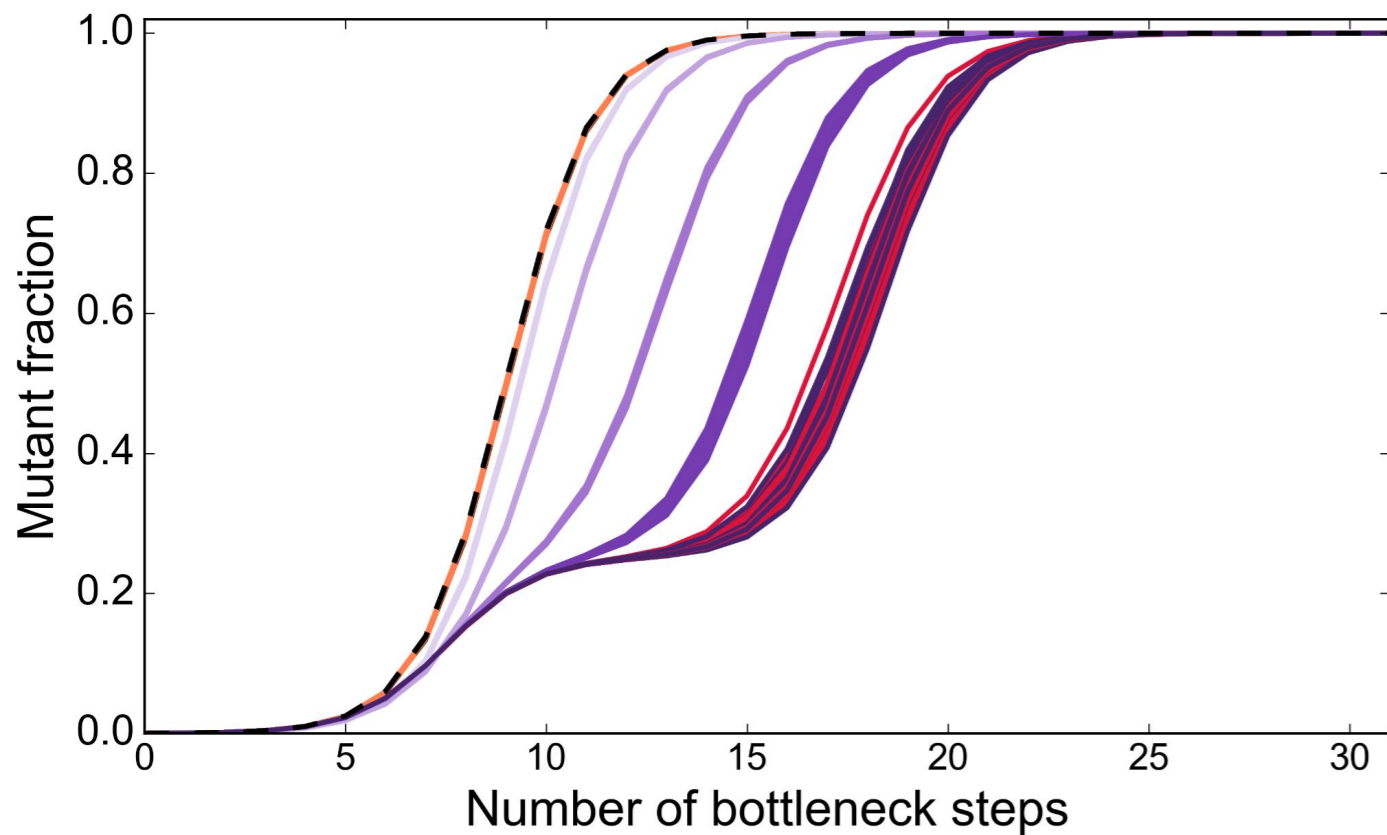

**Mutants initially placed in a leaf**

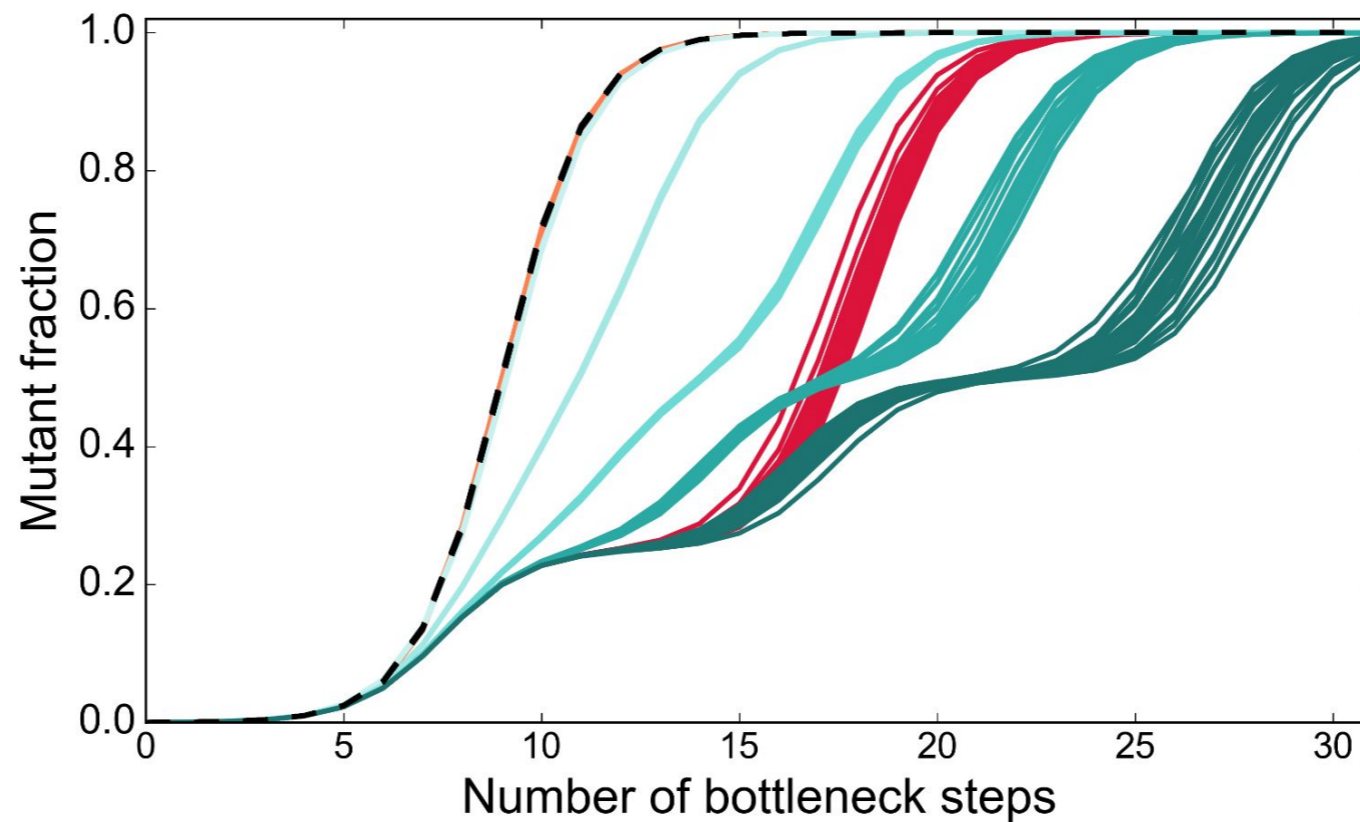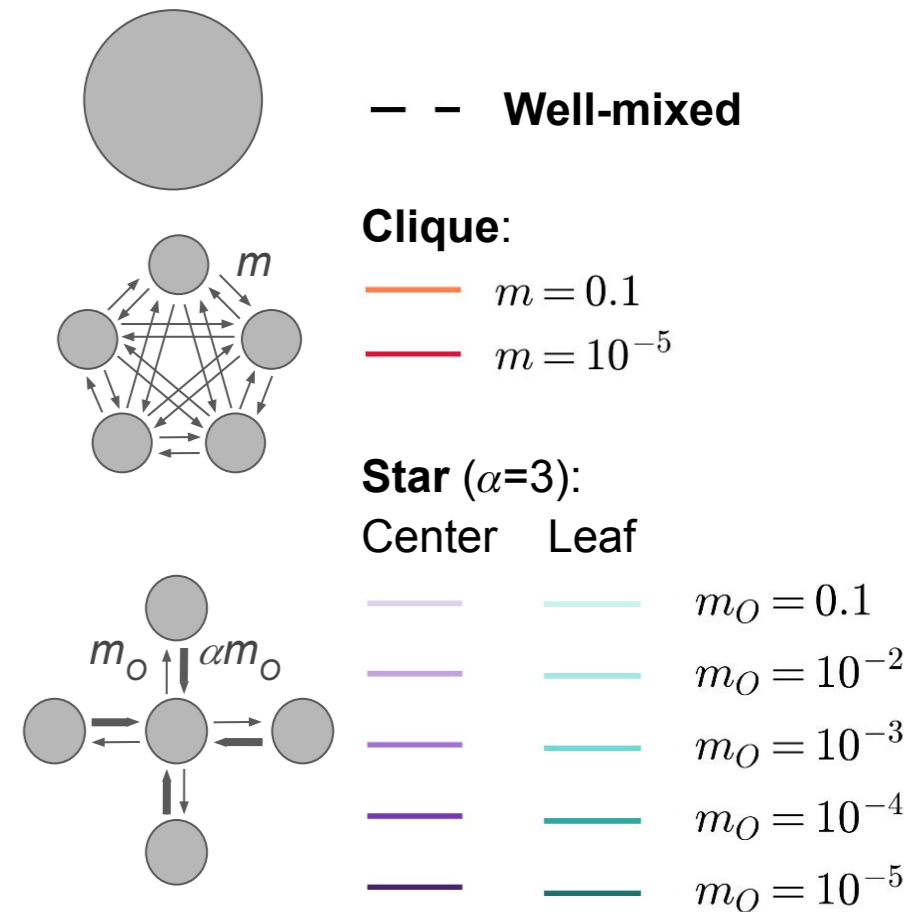

Supplement: S2 Fig — As in Fig 2 and in [35], we consider a clique and a star with D = 4 demes of bottleneck size B = 107 each, where 104 mutants with effective fitness advantage st = 0.2 log(100) are initially placed in one deme. As a reference, we also consider a well-mixed population of total bottleneck size DB = 4 × 10^7^, initialized with 104 mutants. Left: mutants initially placed in the center of the star; right: mutants initially placed in a leaf of the star (the same trajectories are shown in both panels for the clique and the well-mixed population, and serve as references). In each case, we report the mutant fraction versus time (expressed in number of bottleneck steps). We consider the same migration probabilities as in Fig 2. Each line reports the result of a single stochastic simulation (i.e. a single trajectory), and 20 of them are shown in each case. Note that all of them result in mutant fixation. (PDF) [file pcbi.1012424.s003.pdf]

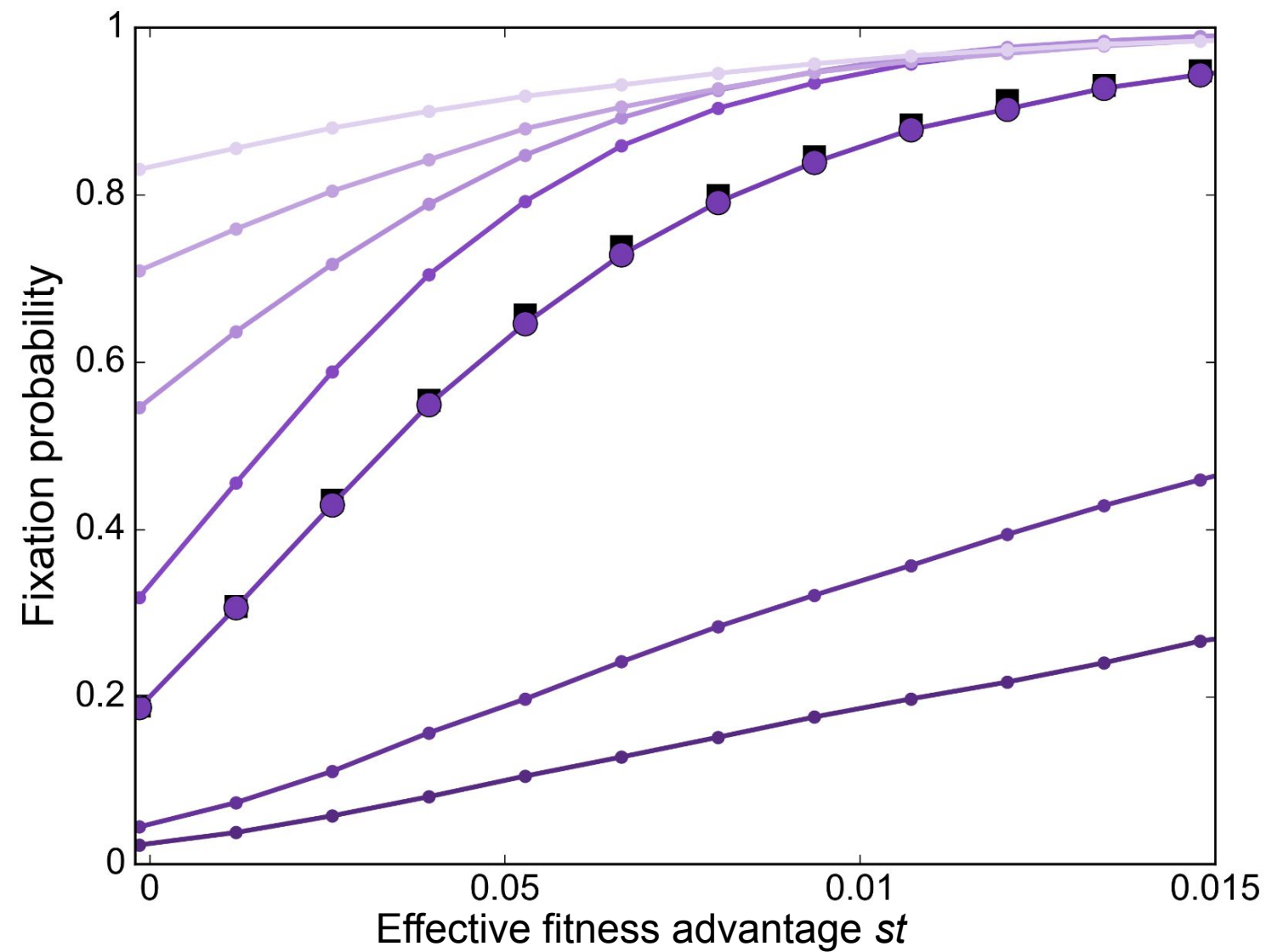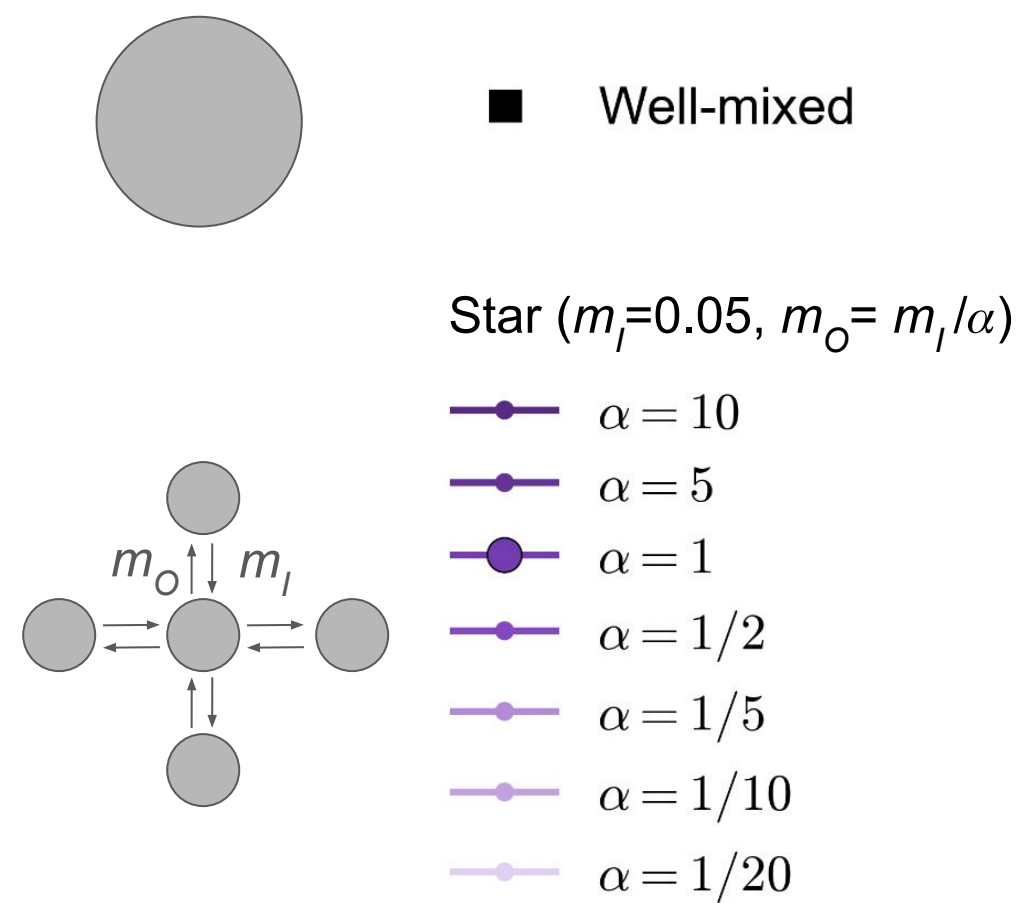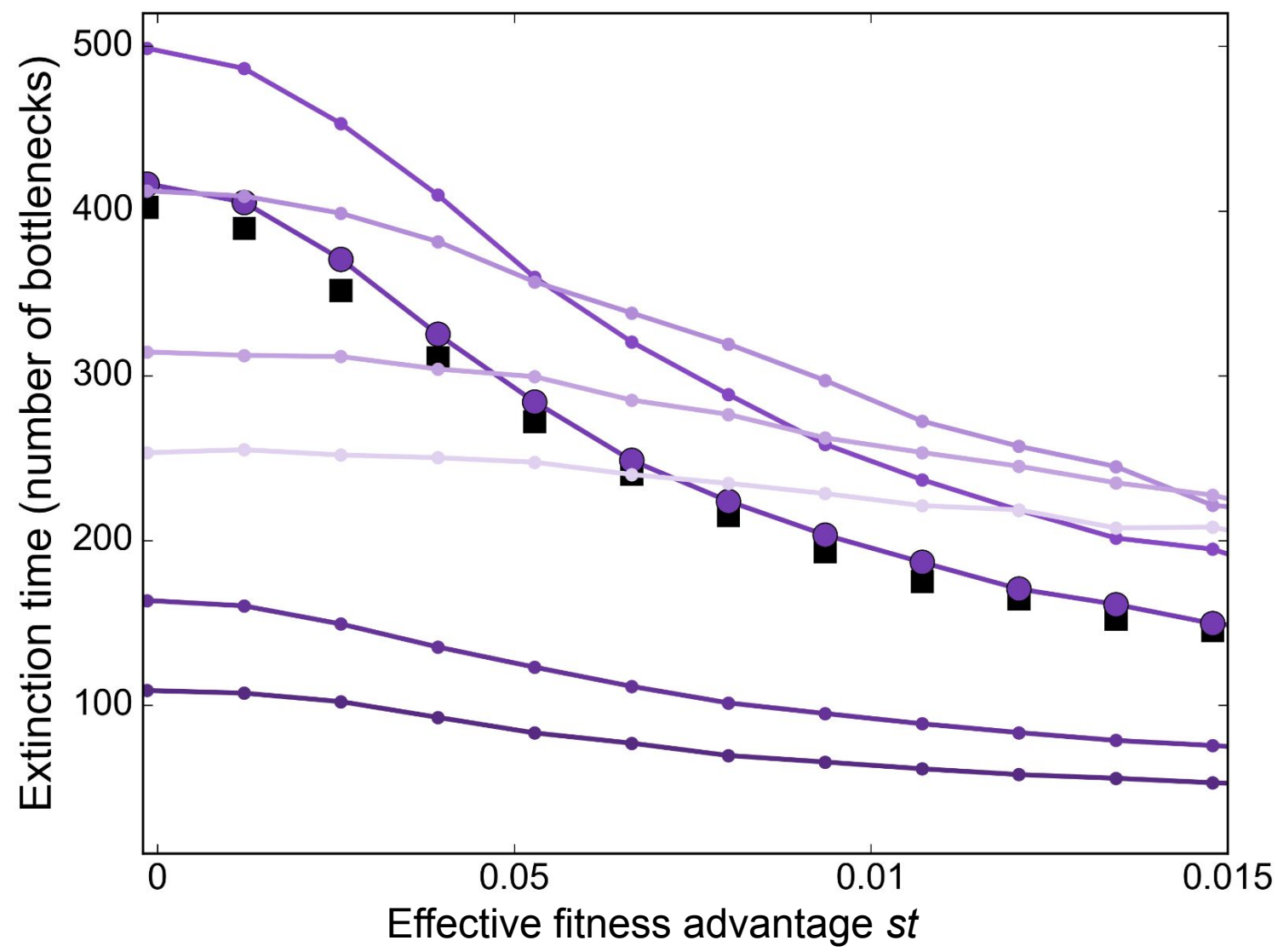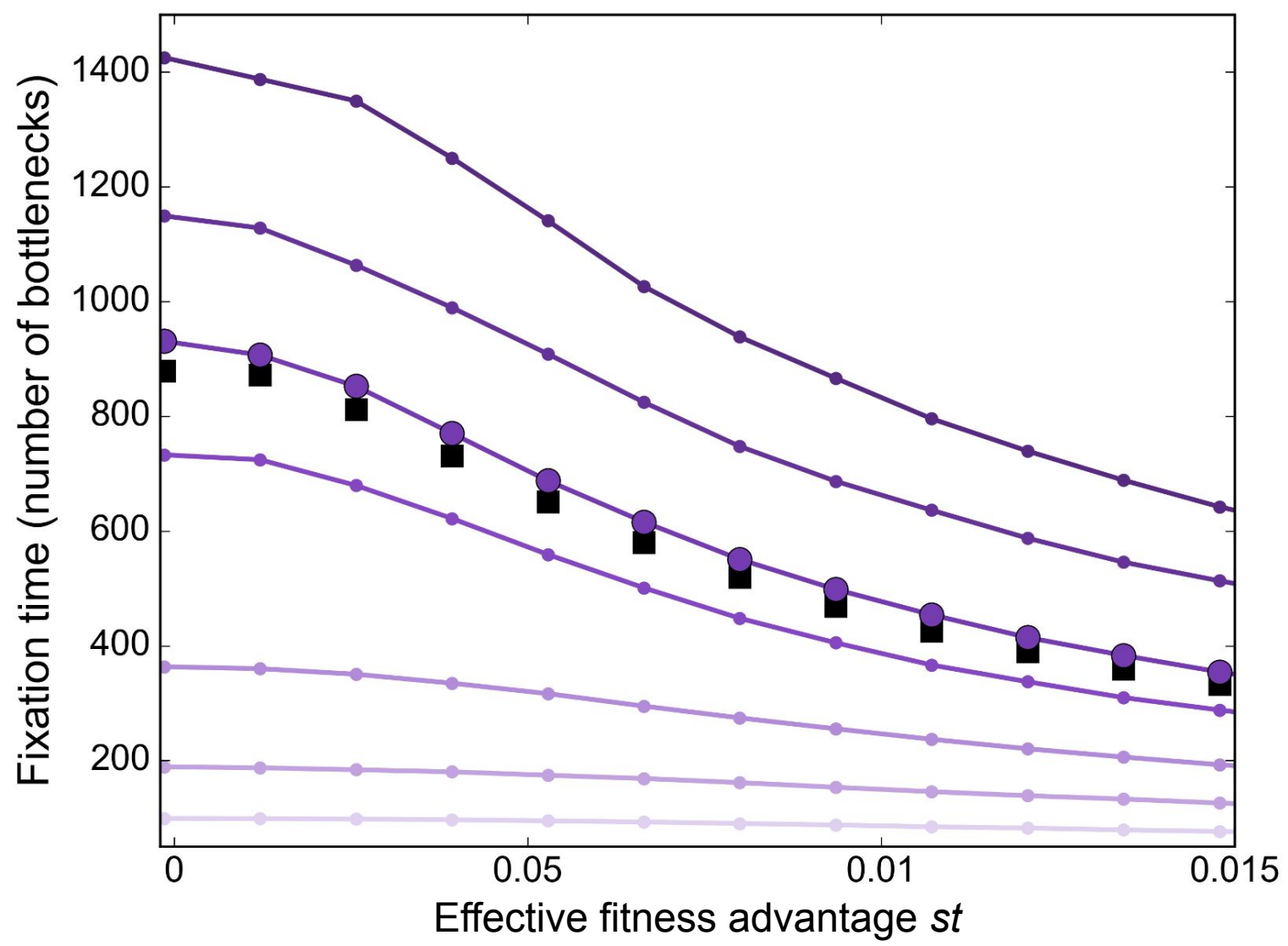

Supplement: S5 Fig — Mutant fixation probability (top), average extinction time (bottom left) and average fixation time (bottom right) are plotted as a function of the effective fitness advantage st of the mutant. We consider a star with D = 5 demes of size B = 100, as in Fig 3, but it is initialized with a fully mutant center. For reference, we also consider a well-mixed population of size DB = 500, initialized with 100 mutants. We take different values of α = mI/mO, always with mI = 0.05, as in Fig 3. Markers are simulation results, obtained over at least 100,000 realizations. Lines linking markers are guides for the eye. (PDF) [file pcbi.1012424.s006.pdf]

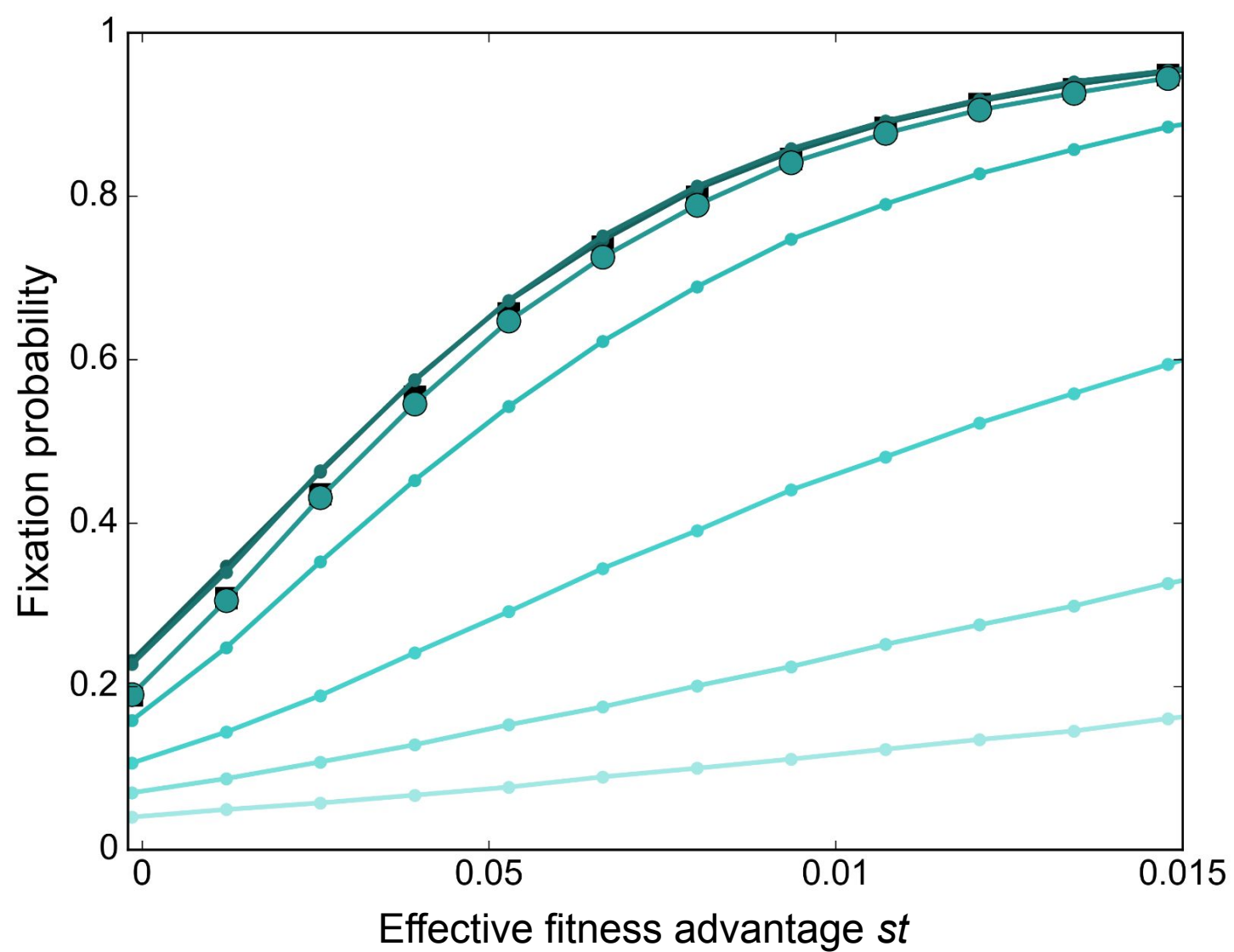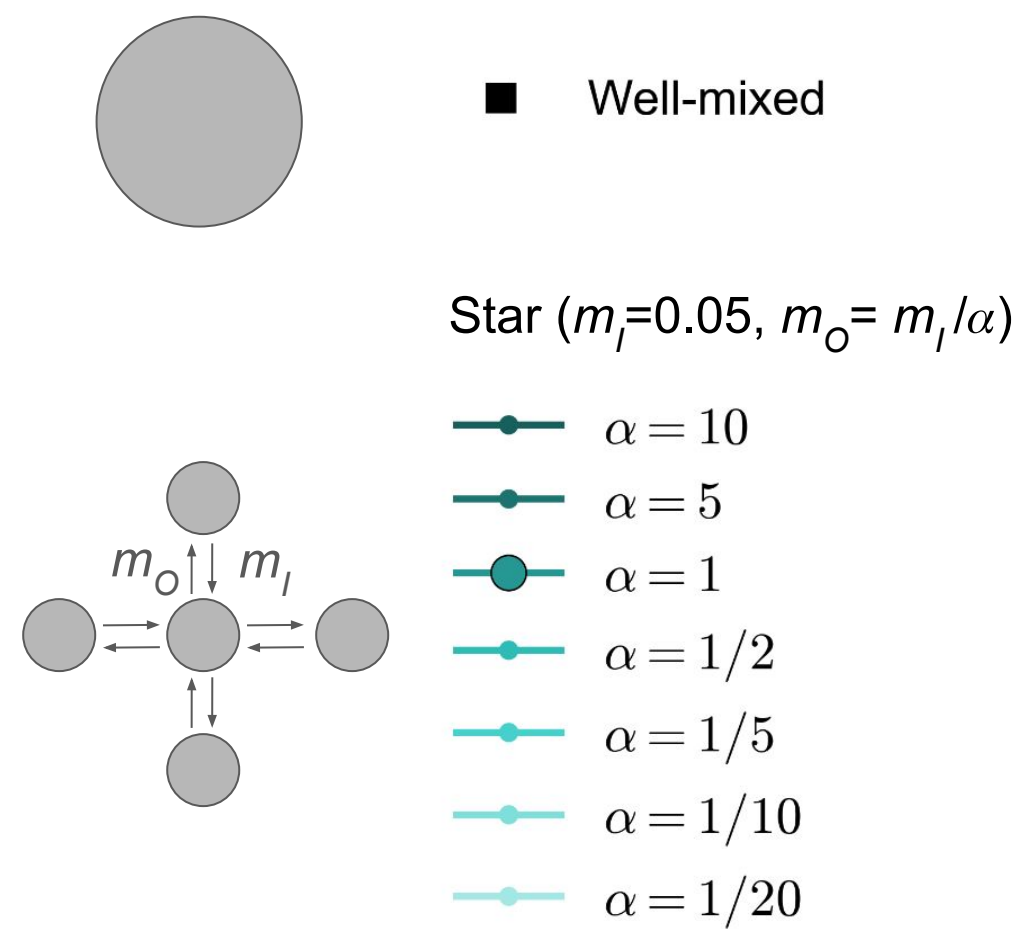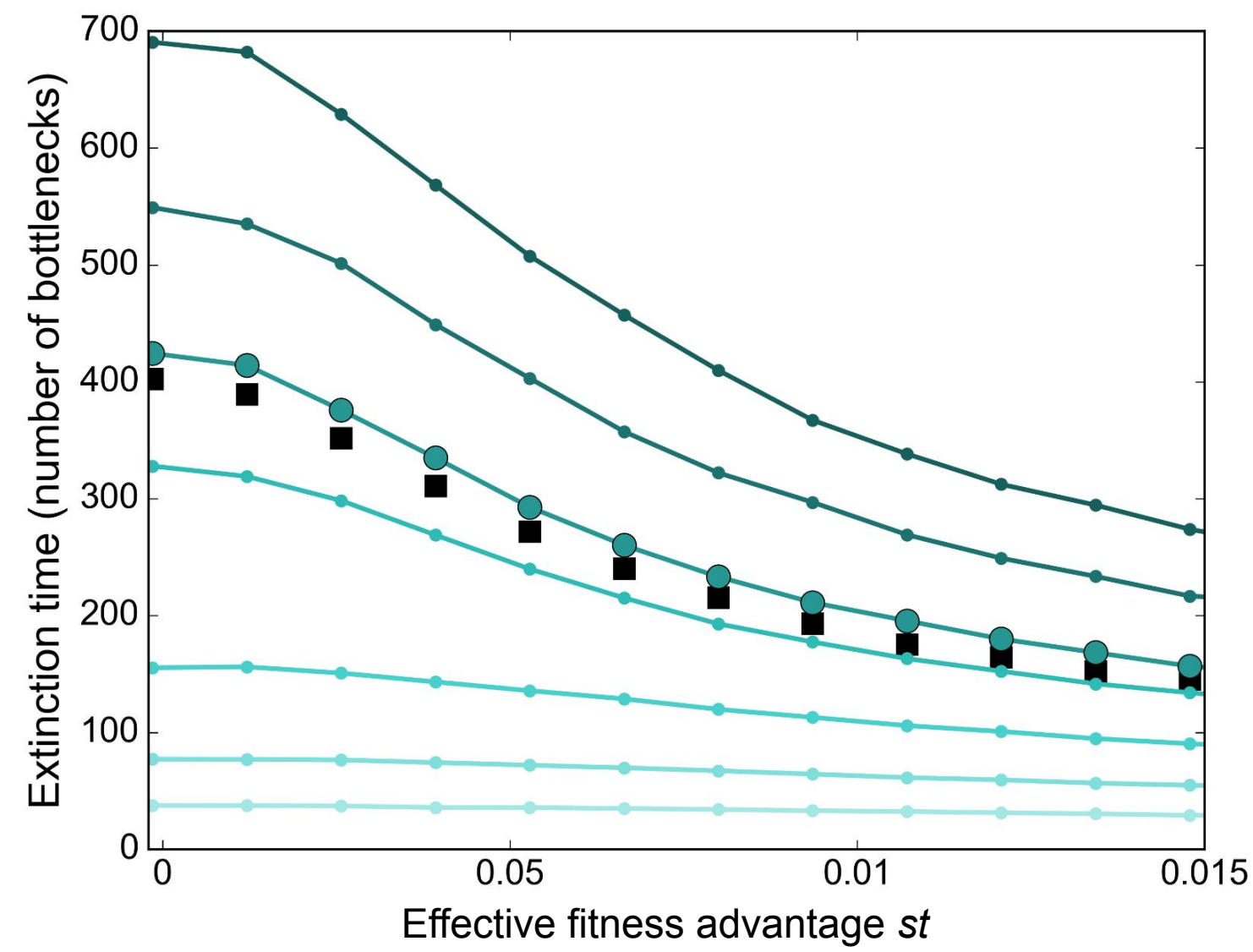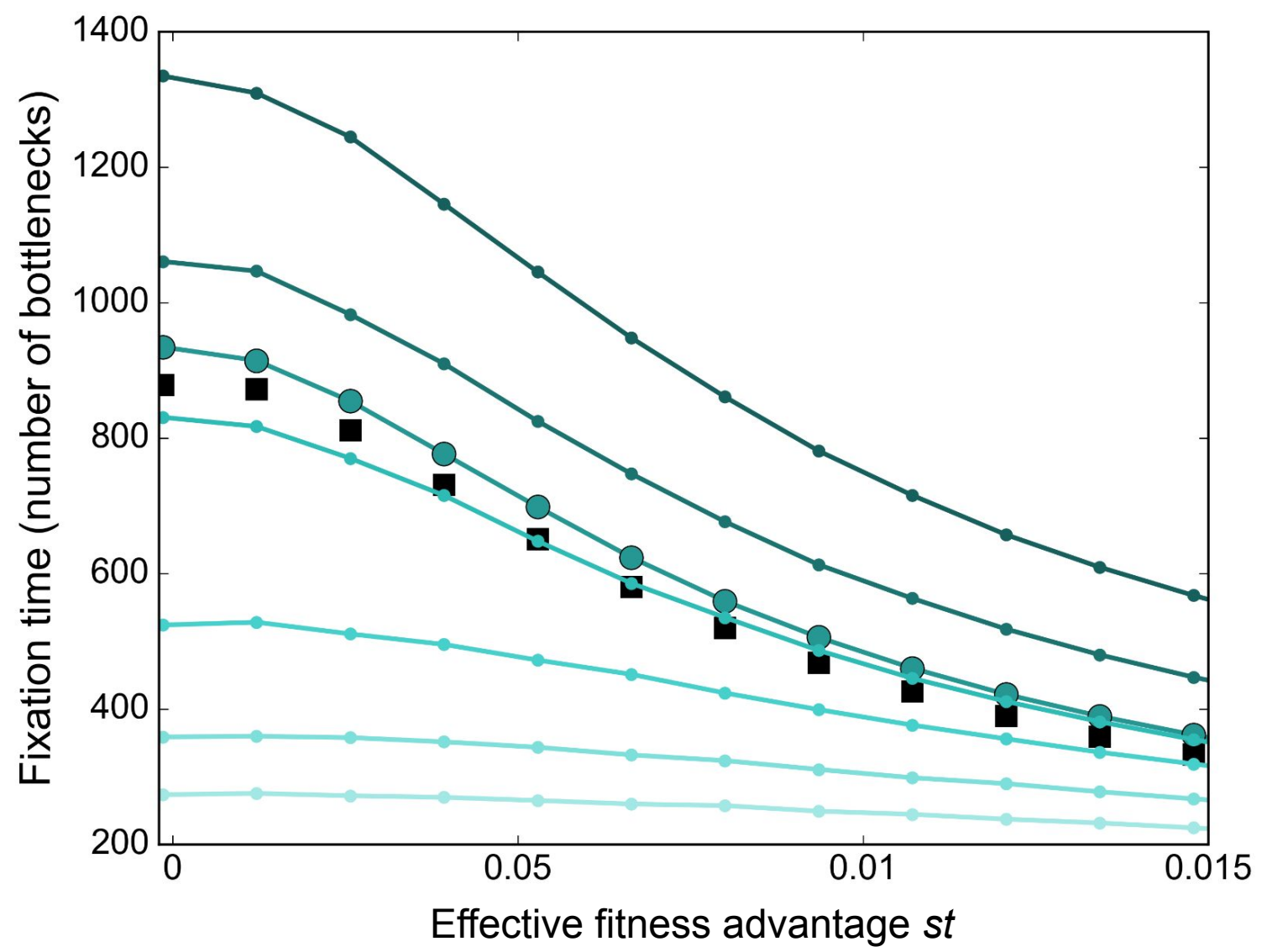

Supplement: S6 Fig — Same as S5 Fig, but starting from a fully mutant leaf instead of a fully mutant center. Mutant fixation probability (top), average extinction time (bottom left) and average fixation time (bottom right) are plotted as a function of the effective fitness advantage st of the mutant. We consider a star with D = 5 demes of size B = 100, as in Fig 3, but it is initialized with a fully mutant leaf. For reference, we also consider a well-mixed population of size DB = 500, initialized with 100 mutants. We take different values of α = mI/mO, always with mI = 0.05, as in Fig 3. Markers are simulation results, obtained over at least 100,000 realizations. Lines linking markers are guides for the eye. (PDF) [file pcbi.1012424.s007.pdf]

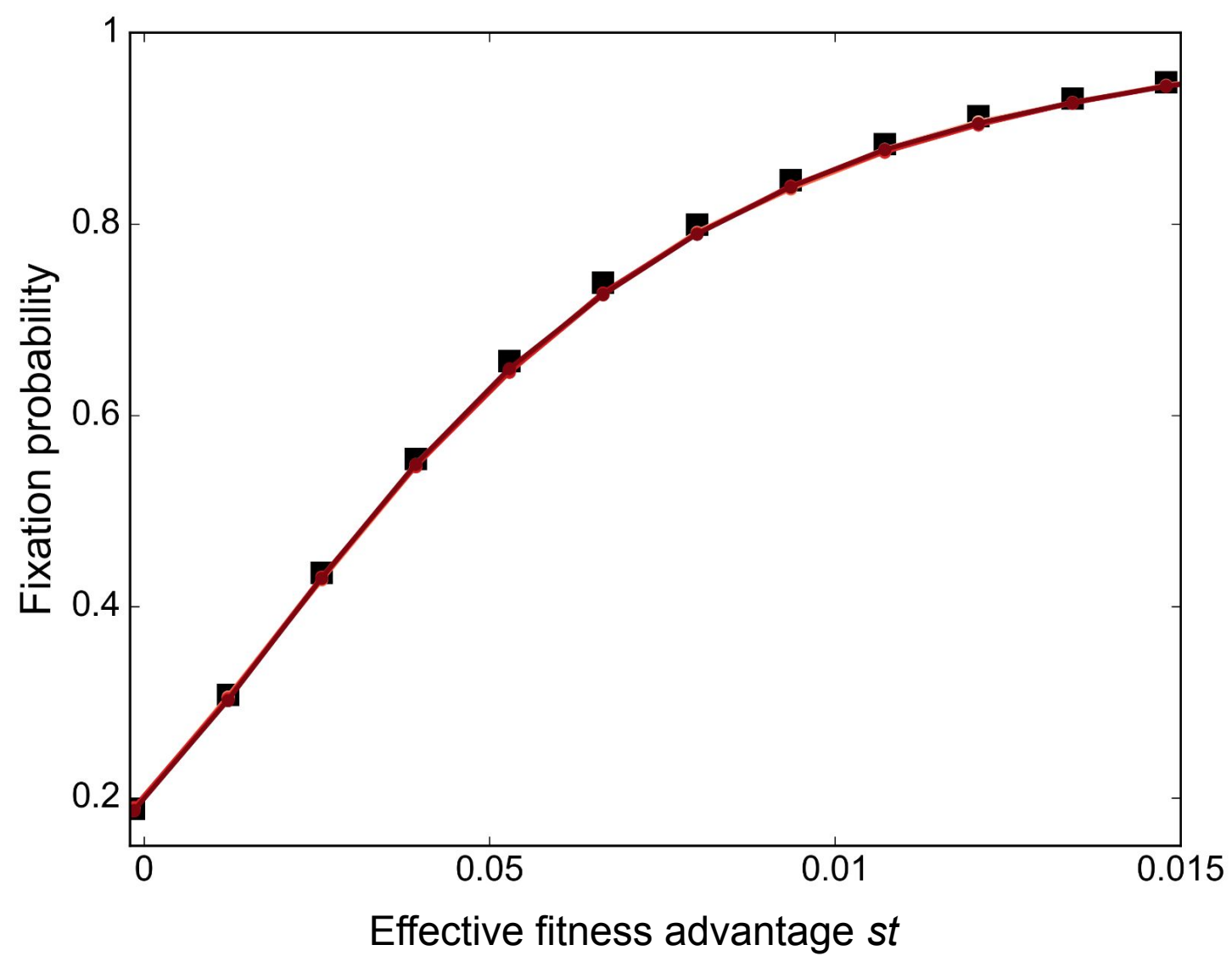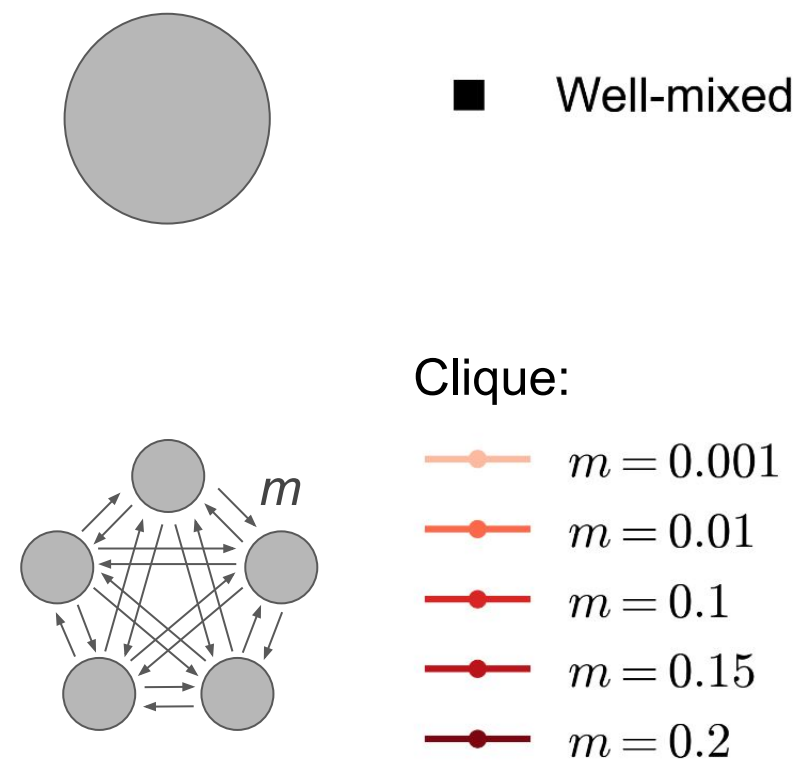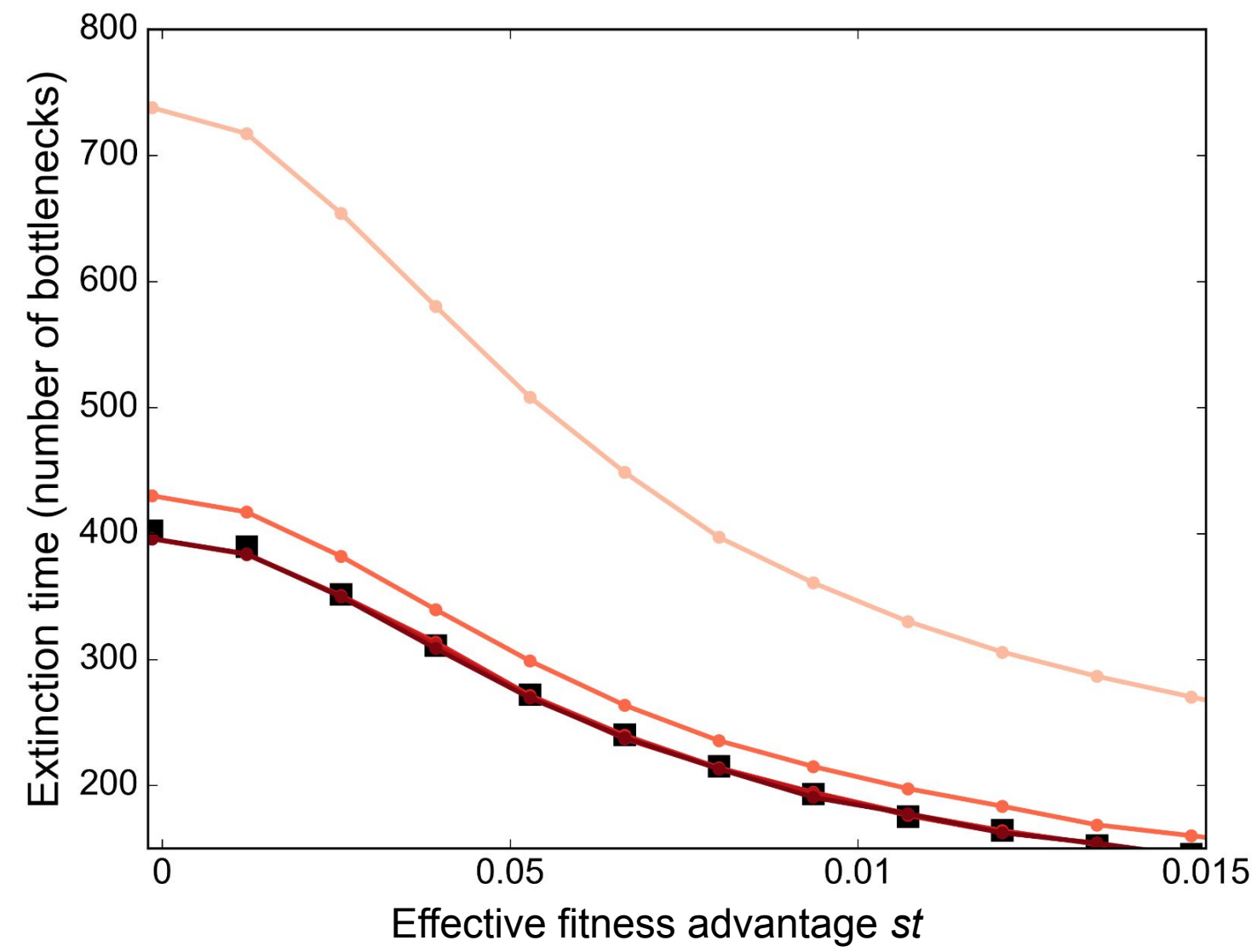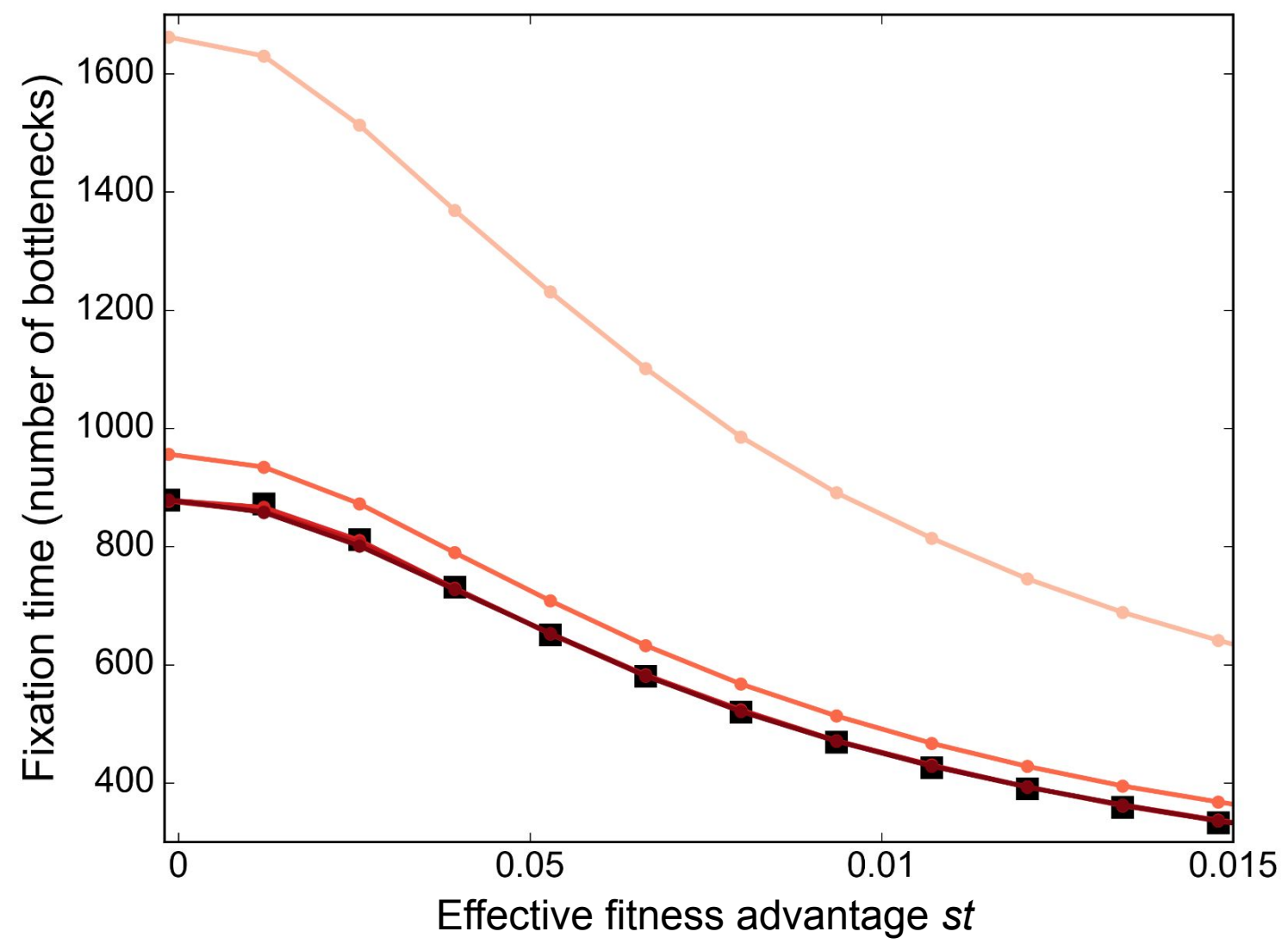

Supplement: S7 Fig — Mutant fixation probability (top), average extinction time (bottom left) and average fixation time (bottom right) are plotted as a function of the effective fitness advantage st of the mutant. We consider a clique with D = 5 demes of size B = 100, initialized with a fully mutant deme, as in Fig 3. For reference, we also consider a well-mixed population of size DB = 500, initialized with 100 mutants. We take different values of the migration probability m. Markers are simulation results, obtained over at least 100,000 realizations. Lines linking markers are guides for the eye. (PDF) [file pcbi.1012424.s008.pdf]
