# Supplementary material for: Mutant fate in spatially structured populations on graphs: Connecting models to experiments
Source: PLoS Comput Biol. 2024 Sep 6;20(9):e1012424. doi: 10.1371/journal.pcbi.1012424 (PMC11410244; doi:10.1371/journal.pcbi.1012424)
Supplement: S3 Fig — As in [35] and in Fig 2, we consider a clique and a star with D = 4 demes of bottleneck size B = 107 each, where 104 mutants with effective fitness advantage st = 0.2 log(100) are initially placed in one deme. This deme is the center (top left), a leaf (top right), or chosen uniformly at random (bottom). As a reference, we also consider a well-mixed population of total bottleneck size DB = 4 × 10^7^, initialized with 104 mutants. In each case, we report the mutant fraction versus time (expressed in number of bottleneck steps). We consider different migration probabilities m for the clique and mI for the star, keeping α = mI/mO = 1/3 for the star (recall that α = 3 in Fig 2). Markers are simulation results averaged over 100 trajectories, and error bars report the standard deviation. Note that all trajectories resulted in mutant fixation. Lines show the predictions from our deterministic model. (PDF) [file pcbi.1012424.s004.pdf]

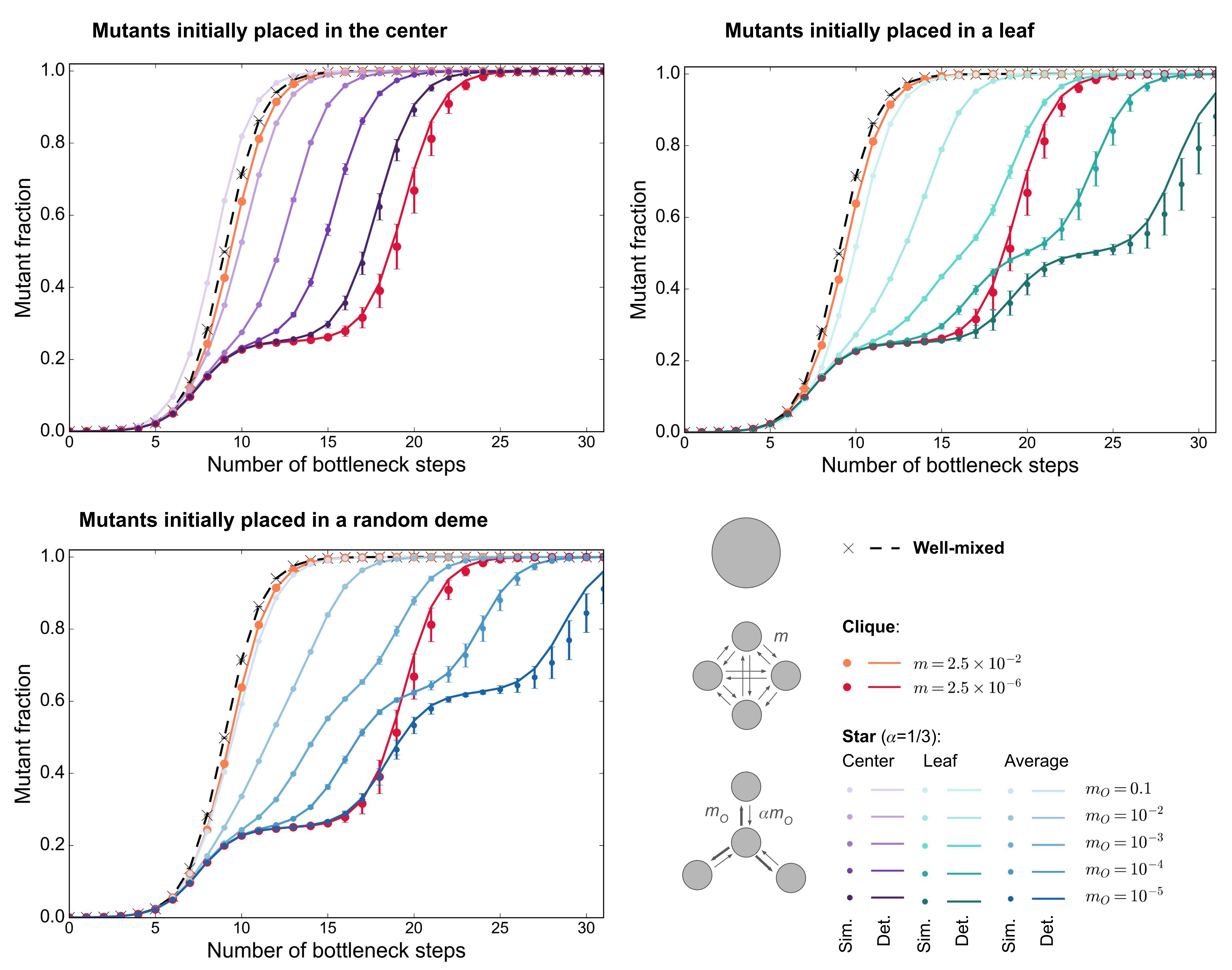

Mutants initially placed in the center
Mutants initially placed in a leaf
Mutants initially placed in a random deme
Mutant fraction
Number of bottleneck steps
0.0
0.2
0.4
0.6
0.8
1.0
0
5
10
15
20
25
30
Well-mixed
Clique:
m
$m = 2.5 \times 10^{-2}$
$m = 2.5 \times 10^{-6}$
Star (α=1/3):
Center
Leaf
Average
m_O
αm_O
$m_O = 0.1$
$m_O = 10^{-2}$
$m_O = 10^{-3}$
$m_O = 10^{-4}$
$m_O = 10^{-5}$
Sim.
Det.
Sim.
Det.
Sim.
Det.
