# Supplementary material for: Mutant fate in spatially structured populations on graphs: Connecting models to experiments
Source: PLoS Comput Biol. 2024 Sep 6;20(9):e1012424. doi: 10.1371/journal.pcbi.1012424 (PMC11410244; doi:10.1371/journal.pcbi.1012424)
Supplement: S4 Fig — Same as Fig 2 and S3 Fig, but for smaller bottleneck deme sizes B = 105, and with α = 3. (PDF) [file pcbi.1012424.s005.pdf]

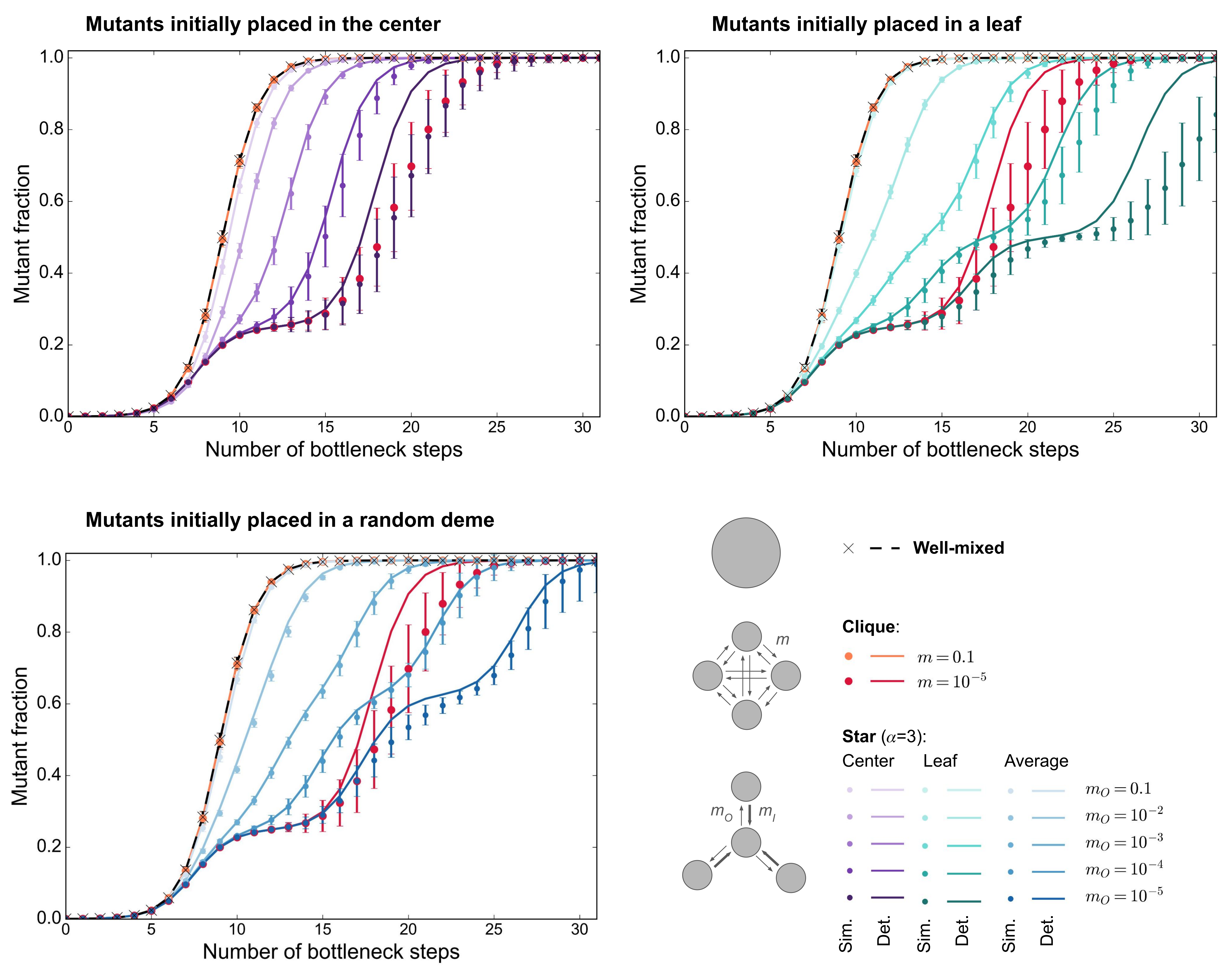

Mutants initially placed in the center
Mutants initially placed in a leaf
Mutants initially placed in a random deme
Mutant fraction
Number of bottleneck steps
Well-mixed
Clique:
m
$m=0.1$
$m=10^{-5}$
Star (α=3):
Center
Leaf
Average
m_O
m_I
$m_O=0.1$
$m_O=10^{-2}$
$m_O=10^{-3}$
$m_O=10^{-4}$
$m_O=10^{-5}$
Sim.
Det.
Sim.
Det.
Sim.
Det.
